# *SLC6A3 *and body mass index in the Prostate, Lung, Colorectal and Ovarian Cancer Screening Trial

**DOI:** 10.1186/1471-2350-10-9

**Published:** 2009-01-30

**Authors:** Elizabeth M Azzato, Lindsay M Morton, Andrew W Bergen, Sophia S Wang, Nilanjan Chatterjee, Paul Kvale, Meredith Yeager, Richard B Hayes, Stephen J Chanock, Neil E Caporaso

**Affiliations:** 1 Division of Cancer Epidemiology and Genetics, National Cancer Institute, NIH, DHHS, Rockville, Maryland, USA; 2 Department of Oncology, University of Cambridge, Strangeways Research Laboratory, Worts Causeway, Cambridge, CB1 8RN, UK; 3 Center for Health Sciences, SRI International, Menlo Park, California, USA; 4 Henry Ford Health System, Detroit, Michigan, USA; 5 Core Genotyping Facility, Division of Cancer Epidemiology and Genetics, Advanced Technology Program, SAIC Frederick, Inc, NCI-Frederick, Frederick, Maryland, USA

## Abstract

**Background:**

To investigate the contribution of the dopamine transporter to dopaminergic reward-related behaviors and anthropometry, we evaluated associations between polymorphisms at the dopamine transporter gene(*SLC6A3*) and body mass index (BMI), among participants in the Prostate, Lung, Colorectal and Ovarian (PLCO) Cancer Screening Trial.

**Methods:**

Four polymorphisms (rs6350, rs6413429, rs6347 and the 3' variable number of tandem repeat (3' VNTR) polymorphism) at the *SLC6A3 *gene were genotyped in 2,364 participants selected from the screening arm of PLCO randomly within strata of sex, age and smoking history. Height and weight at ages 20 and 50 years and baseline were assessed by questionnaire. BMI was calculated and categorized as underweight, normal, overweight and obese (<18.5, 18.5–24.9, 25.0–29.9, or ≥ 30 kg/m^2^, respectively). Odds ratios (ORs) and 95% confidence intervals (CIs) of *SLC6A3 *genotypes and haplotypes were computed using conditional logistic regression.

**Results:**

Compared with individuals having a normal BMI, obese individuals at the time of the baseline study questionnaire were less likely to possess the *3' *VNTR variant allele with 9 copies of the repeated sequence in a dose-dependent model (** is referent; OR_*9 _= 0.80, OR_99 _= 0.47, p_trend _= 0.005). Compared with individuals having a normal BMI at age 50, overweight individuals (A-C-G-* is referent; OR_A-C-G-9 _= 0.80, 95% CI 0.65–0.99, p = 0.04) and obese individuals (A-C-G-* is referent; OR_A-C-G-9 _= 0.70, 95% CI 0.49–0.99, p = 0.04) were less likely to possess the haplotype with the 3'variant allele (A-C-G-9).

**Conclusion:**

Our results support a role of genetic variation at the dopamine transporter gene, *SLC6A3*, as a modifier of BMI.

## Background

Modifiable behavioral risk factors, specifically poor diet and physical inactivity, tobacco use and alcohol consumption are major causes of mortality in the United States [1]. Family, twin and adoption studies provide evidence that inherited variation makes a substantial contribution to body fat/obesity, smoking and alcohol dependence. With regard to obesity, the heritability of BMI is estimated to be significant and to range up to 80% in large twin samples [2,3]

Nearly two-thirds of the US population is overweight or obese, and increased adiposity increases risk of death from heart disease, type 2 diabetes mellitus, hypertension, and diverse cancers [4]. Mutations in leptin (*LEP*), prohormone convertase 1 (*PCSK1*), proopiomelanocortin (*POMC*) and melanocortin 4 receptor (*MC4R*) are involved in rare monogenic recessive forms of obesity [5], but studies relying on populations with morbid obesity may not represent processes contributing to overweight and obesity in the general population. The genetic variation that contributes to susceptibility to obesity in the general population is beginning to be elucidated through candidate gene and genome wide studies focused on obesity and related phenotypes [6]. Recent genome wide association scans have identified regions in the *FTO *gene [7-10] and variants telomeric to the *MC4R *gene to be associated with obesity [11,12]. The genes identified to date likely only identify a small proportion of the genetic variability that influences susceptibility to obesity.

Diverse lines of evidence implicate heritable differences in the dopamine reward pathway associated with the consumption of food, as well as alcohol dependence and tobacco use [13-21]. Dopamine is a key neurotransmitter mediating reward, and therefore, it is relevant to diverse behavioral conditions including obesity. *SLC6A3*, the dopamine transporter gene, is an important polymorphic gene which controls the reuptake of dopamine in the synapse, in areas of the CNS crucial to reward and learning such as the mesolimbic pathway. Early genetic association studies in the general population examined *SLC6A3 *in relation to smoking and alcohol intake [22,23], and there is evidence for a relationship of *SLC6A3 *genotype to BMI in a small study in African Americans [24]. However, a large, representative United States population has not been studied to date.

We previously reported an association with the *DRD2 *TaqI and IVS6-83 variant alleles and the wild-type-239 allele with obesity [25,26]. In order to further evaluate the effects of common inherited variation in the dopamine pathway on BMI, we extended our study to include four polymorphisms in the gene that codes for the dopamine transporter (DAT1), *SLC6A3*, among 2,364 participants in the Prostate, Lung, Colorectal, and Ovarian (PLCO) Cancer Screening Trial.

## Methods

### Study population

The PLCO Cancer Screening Trial study population has been described previously [27]. Briefly, over 150,000 individuals aged 55–74 years from ten US study centers were randomized during 1992–2001 to undergo a specific cancer screening regimen or receive routine medical care to evaluate the effects of screening on disease-specific mortality. At entry into the trial, participants provided written, informed consent and completed a baseline demographic and risk factor questionnaire. Participants in the screening arm were also asked to provide separate written, informed consent and a blood sample for use in etiologic studies.

Adult height and weight at baseline and ages 20 and 50 were obtained from the baseline questionnaire. Body mass index (BMI, kg/m^2^) was computed. Individuals were characterized at each age as underweight, normal weight, overweight, or obese. Information on the smoking habits of smokers and alcohol consumption were obtained in the baseline questionnaire; non-smokers were defined as individuals who had never smoked cigarettes regularly for at least six months. Additional information on race/ethnicity, highest level of schooling completed and marital status were also obtained from the baseline questionnaire.

### Design

We originally selected a sample for investigations of behavioral characteristics (smoking, alcohol dependence and BMI) related to cancer, and we have reported the smoking results [25,28]. The sample consisted of 2,406 participants selected randomly after stratifying by sex, age (55–59, 60–64, 65–69, 70–74 years), smoking status (never, current, former), and quantity of cigarettes smoked for current and former smokers (1–10, 11–20, 21+ cigarettes per day). Blood samples with sufficient DNA for genotyping were available for 2,379 (98.9%) of these individuals. An additional 15 individuals were excluded due to missing data for BMI at all three time points (ages 20 and 50 years and baseline), yielding a final analytic population of 2,364 participants. This study was conducted according to a protocol approved by the Institutional Review Board of the National Cancer Institute.

### Genotyping

We selected for genotyping three single nucleotide polymorphisms (SNPs) and one variable number of tandem repeat (VNTR) polymorphism in *SLC6A3 *(see Additional file 1, Supplemental table 1). Two of the selected SNPs, *SLC6A3 *1215A>G (Ex9-55; S405S) and *SLC6A3 *114C>T (Ex2+159; N38N) are in the coding region of *SLC6A3*. The third SNP, *SLC6A3 *-3714G>T, is located in the 5' non-coding region. With respect to the *SLC6A3 *VNTR genotype, the * symbol designates any VNTR allele other than the 9 allele. We also evaluated four SNPs in *DRD2*/*ANKK1*, which have been previously reported [25] for a possible gene-gene effect (see Additional file 1, Supplemental table 1). All polymorphisms were chosen based on biologic plausibility, previous research, availability of functional data, potential linkage disequilibrium with other functional SNPs, and minor allele frequency greater than 5% [29]. DNA extraction and genotyping and quality control data are reported elsewhere [25].

**Table 1:** Selected characteristics of study participants from the Prostate, Lung, Colorectal and Ovarian Cancer Screening Trial (PLCO)

	Current BMI*			
	
	Underweight	Normal	Overweight	Obese
	N = 26	N = 898	N = 989	N = 443
Gender, # (%)				
Male	4 (15.4)	380 (42.3)	571 (57.7)	232 (52.4)
Female	22 (84.6)	518 (57.7)	418 (42.3)	211 (47.6)
				
Age in yrs, # (%)				
50–59	3 (11.5)	218 (24.2)	248 (25.1)	126 (28.4)
60–64	7 (26.9)	194 (21.6)	268 (27.1)	128 (28.9)
65–69	8 (30.8)	254 (28.3)	258 (26.1)	108 (24.4)
70–74	8 (30.8)	232 (25.8)	215 (21.7)	81 (18.3)
				
Race, # (%)				
Caucasian	23 (88.5)	799 (89.0)	888 (89.8)	359 (81.0)
African American	1 (3.8)	37 (4.1)	43 (4.3)	30 (6.7)
Other	2 (7.7)	62 (6.9)	58 (5.9)	18 (4.1)
				
Education, # (%)				
<= 12 years	14 (53.8)	264 (29.4)	306 (31.0)	165 (37.3)
Some College	7 (26.9)	318 (35.5)	376 (38.1)	167 (37.7)
College grad.	5 (19.2)	315 (35.1)	305 (30.9)	110 (24.9)
Missing	0 (0.0)	1 (0.002)	2 (0.002)	1 (0.003)
				
Marital Status, # (%)				
Married/living as married	14 (53.8)	613 (68.3)	716 (72.4)	313 (70.7)
Unmarried	12 (46.2)	285 (31.7)	271 (27.4)	103 (23.3)
Missing	0 (0.0)	0 (0.0)	2 (0.002)	0 (0.0)
				
Smoking Status, # (%)				
Never	3 (11.5)	81 (9.0)	84 (8.5)	43 (9.7)
Current	19 (73.1)	461 (51.3)	434 (43.9)	146 (33.0)
Former	4 (15.4)	356 (39.6)	471 (47.6)	254 (57.3)
				
Cigarettes per day, # (%)				
1–10	8 (34.8)	286 (35.0)	286 (31.6)	135 (33.8)
11–20	9 (39.1)	286 (35.0)	316 (34.9)	111 (27.8)
21+	6 (26.1)	245 (30.0)	303 (33.5)	154 (38.5)

* BMI categorized as Underweight (≤ 18 kg/m^2^), Normal (18–24.9 kg/m^2^), Overweight (25–29.9 kg/m^2^) or Obese (≥ 30 kg/m^2^); data on BMI at baseline were missing for N = 8 individuals.

### Statistical analysis

Hardy-Weinberg equilibrium was evaluated among non-Hispanic Caucasians for each polymorphism by computing the Pearson chi-square statistic. Haplotypes were visualized using Haploview, version 3.11, based on measures of pairwise linkage disequilibrium between polymorphisms. SAS/Genetics, version 8.2 (SAS Institute, Inc., Cary, NC), was used to generate maximum likelihood estimates of haplotype frequencies and to assign the most probable haplotypes for each individual. Haplotypes with an estimated frequency of greater than 1% were considered in our analyses. Individuals with missing values for one or more genotypes were excluded from the haplotype analyses for that gene. As haplotype analyses are potentially more sensitive to ethnic variation, these analyses were limited to non-Hispanic Caucasians only. The probability of the assigned haplotype pair was greater than 99% for approximately 80% of individuals.

Individuals were characterized at each age as underweight, normal weight, overweight, or obese (<18.5, 18.5–24.9, 25.0–29.9, or ≥ 30 kg/m^2^, respectively). Change in weight over time was assessed in two ways. First, individuals were categorized into four groups based on percent change in weight from age 20 to age 50 and from age 20 to current age (≤ 10%, 10.1–20.0%, 20.1–30.0%, or > 30.0%). Second, change in weight per 10 years was computed by regressing BMI at ages 20, 50 and age at a baseline measurement recorded at study enrollment against age. The slope of each regression line was then characterized into four groups (≤ 0.30, 0.31–0.70, 0.71–1.20, or > 1.20 kg/m^2 ^per 10 years). This slope of BMI change corresponds to a change in weight, as height was only reported once at the time of baseline questionnaire. Intensity of cigarette smoking was categorized as light, medium, or heavy (1–10, 11–20, or ≥ 21 cigarettes per day, respectively).

Cross sectional analysis was conducted to compare individuals by behavioral characteristics at baseline (obese, overweight, or underweight versus normal weight individuals, smoking status and alcohol use), to compare individuals by percent change in weight from age 20 to current age (10.1–20.0%, 20.1–30.0% or > 30.0% versus ≤ 10%) and finally to compare individuals by BMI slope, the change in weight per 10 years (0.31–0.70, 0.71–1.20, or > 1.20 versus ≤ 0.30 kg/m^2 ^per 10 years).

The sample was originally selected to examine behavioral characteristics relevant to cancer (smoking, alcohol use and BMI), so data had been sampled by categories of current smoking status and cigarettes per day, age group and gender. Differences between comparison groups in the distribution of *SCL6A3 *gene variants were quantified using multivariate conditional logistic regression models, conditioned on the variables used to construct the stratified sample (sex, age, smoking status, and quantity of cigarettes smoked for current and former smokers). Conditional logistic regression was necessary, as the original study design involved sampling according to these variables. In such a case, conditional logistic regression most appropriately models the sampling frame to obtain proper risk estimates. Attempts to conduct an unconditional logistic regression including all strata terms resulted in non-convergence of the model.

Odds ratios (ORs) and 95% confidence intervals (CIs) were computed for individual genotypes with the common allele homozygote as the referent. In the case of haplotypes, data were analyzed with the haplotype containing the most common allele for each locus as the referent. Using an additive (dose-dependent) model, we also computed a p-value for the linear trend based on a three-level variable for each genotype (common allele homozygote, heterozygote, variant allele homozygote); this is equivalent to a dose-dependent effect with regard to the number of copies of the variant allele. Finally, adjusted analyses on baseline BMI and *SLC6A3 *genotype were conducted stratified by *DRD2 *haplotype, to evaluate a possible interaction between *DRD2 *and *SLC6A3*.

All models were also adjusted for self-identified race/ethnicity (Caucasian, African American, other). Inclusion of additional demographic factors, such as alcohol use, education, and marital status, in the models did not substantially modify the parameter estimates (± 10%) and were therefore excluded from the model. The SAS System, version 9.1 (SAS Institute, Inc., Cary, NC), was used to conduct all statistical analyses. P-values are presented with one significant digit. Statistical tests were two-sided with an α-level of 0.05.

## Results

Genotype concordance rate was greater than 97%. We observed no significant deviation from Hardy-Weinberg equilibrium for any of the polymorphisms (all p > 0.80).

Underweight, normal weight, overweight, and obese individuals differed significantly by gender (p < 0.0001), age (p = 0.006), education (p = 0.0008), smoking status (p < 0.0001) and cigarettes per day (among smokers, p = 0.04) (Table 1). Specifically, obese individuals tended to be male, younger, less highly educated, married, and former smokers.

BMI was significantly associated with *SLC6A3 *polymorphisms and haplotypes (Table 2). Compared with individuals with normal BMI at baseline, obese individuals (BMI ≥ 30 kg/m^2^) were less likely to possess the *SLC6A3 *VNTR variant allele (9 copies of the VNTR) in a dose-dependent model (** is referent; OR_*9 _= 0.80, OR_99 _= 0.47, p_trend _= 0.005). Consistent with the single polymorphism analyses, obese individuals at baseline were less likely than individuals with normal BMI to possess the haplotype with the *SLC6A3 *VNTR variant allele (A-C-G-9) (A-C-G-* is referent; OR_A-C-G-9 _= 0.60, 95% CI 0.45–0.80, p = 0.001). Results were consistent for the analysis at age 50. Compared with individuals with normal BMI at age 50, overweight individuals (A-C-G-* is referent; OR_A-C-G-9 _= 0.80, 95% CI 0.65–0.99, p = 0.04) and obese individuals (A-C-G-* is referent; OR_A-C-G-9 _= 0.70, 95% CI 0.49–0.99, p = 0.04) were less likely to possess the haplotype with the *SLC6A3 *variant allele (A-C-G-9). No significant trends were noted at age 20.

**Table 2:** *SLC6A3 *polymorphisms and haplotypes by body mass index

	**Normal**	**Underweight BMI**†				**Overweight BMI**†				**Obese BMI**†			
	
**Polymorphism**	**N**	**N**	**OR**‡	**(95% CI)**	**P**	**N**	**OR**‡	**(95% CI)**	**P**	**N**	**OR**‡	**(95% CI)**	**P**
SLC6A3 VNTR													
**	459	14	1.00	(reference)		514	1.00	(reference)		254	1.00	(reference)	
*9	361	12	1.07	(0.48, 2.38)	0.870	390	0.93	(0.76, 1.13)	0.459	159	0.80	(0.62, 1.04)	0.093
99	67	0	~			63	0.81	(0.56, 1.19)	0.284	19	**0.47**	**(0.27, 0.83)**	**0.009**
*P for trend*								*p = 0.246*				***p = 0.005***	
*9/99			0.91	(0.41, 2.01)	0.809		0.91	(0.75, 1.10)	0.332		**0.75**	**(0.59, 0.96)**	**0.023**
Ex9-55A>G													
AA	498	8	1.00	(reference)		539	1.00	(reference)		220	1.00	(reference)	
AG	338	14	**2.66**	**(1.08, 6.58)**	**0.034**	369	1.03	(0.85, 1.25)	0.771	193	**1.34**	**(1.04, 1.73)**	**0.023**
GG	59	4	**6.12**	**(1.66, 22.61)**	**0.007**	80	1.25	(0.86, 1.82)	0.234	27	0.99	(0.60, 1.66)	0.981
*P for trend*				***p = 0.003***				*p = 0.329*				*p = 0.152*	
AG/GG			**3.04**	**(1.28, 7.24)**	**0.012**		1.06	(0.88, 1.28)	0.533		**1.29**	**(1.01, 1.65)**	**0.042**

**Haplotype **§													

A-C-G-*	795	22	1.00	(reference)		906	1.00	(reference)		419	1.00	(reference)	
A-C-G-9	251	3	0.44	(0.13, 1.49)	0.187	252	0.85	(0.69, 1.05)	0.126	84	**0.60**	**(0.45, 0.80)**	**0.001**
A-T-T-*	86	0	~			81	0.90	(0.65, 1.25)	0.529	34	0.79	(0.51, 1.23)	0.298
G-C-G-*	213	10	1.84	(0.83, 4.05)	0.132	242	1.03	(0.83, 1.28)	0.761	124	1.15	(0.88, 1.50)	0.319
G-C-G-9	173	7	1.51	(0.62, 3.70)	0.369	200	1.00	(0.79, 1.26)	0.988	75	0.88	(0.64, 1.20)	0.408

* = an allele other than the SLC6A3*9 allele. These are largely (98.1%) the SLC6A3*10 VNTR allele.

† BMI categorized as Underweight (≤ 18 kg/m^2^), Normal (18–24.9 kg/m^2^), Overweight (25–29.9 kg/m^2^) or Obese (≥ 30 kg/m^2^).

‡ OR estimated using conditional logistic regression, conditioning on age, sex, current smoking status, number of cigarettes smoked.

§Haplotype analyses were conducted among non-Hispanic Caucasians.

Compared with individuals having a normal BMI, underweight individuals (BMI < 18.5 kg/m^2^) at the time of the baseline study questionnaire were significantly more likely to possess the variant allele for the *SLC6A3 *polymorphism Ex9-55A>G in a dose-dependent model (AA is referent; OR_AG _= 2.7, OR_GG _= 6.1, p_trend _= 0.003) (Table 2). Results from the haplotype analyses were consistent with those from the single polymorphism analyses (Table 2).

Percent change in weight and weight change slope were also associated with *SLC6A3 *haplotypes, although the association between individual polymorphisms and percent change in weight did not achieve statistical significance. Individuals who carried the 9 VNTR allele on a wild-type background for the other SNPs (A-C-G-9) were less likely to significantly increase their weight from age 20 to age 50 (Table 3). Similarly, individuals with this haplotype were less likely to experience rapid weight gain (increased slope of weight change) from age 20 to the time of the baseline questionnaire (see Additional file 1, Supplemental table 2).

**Table 3:** SLC6A3 polymorphisms and haplotypes by percent change in weight

				**% Change in weight from age 20 to age 50**					
				
				**5.1–15.0% (N = 806) vs. ≤ 5% (N = 515)**		**15.1–25.0% (N = 524) vs. ≤ 5% (N = 515)**		**> 25% (N = 481) vs. ≤ 5% (N = 515)**	
**Polymorphism**		**N**	**G Freq (%)**	**OR**‡	**(95% CI)**	**OR**‡	**(95% CI)**	**OR**‡	**(95% CI)**

SLC6A3 VNTR	*9	724	39.9	0.88	(0.70, 1.13)	0.92	(0.71, 1.20)	0.90	(0.68, 1.18)
	99	119	6.5	1.10	(0.70, 1.73)	0.70	(0.41, 1.22)	0.64	(0.36, 1.14)
Ex9-55A>G	AG	711	39.0	1.16	(0.91, 1.48)	1.28	(0.98, 1.67)	1.14	(0.87, 1.50)
	GG	138	7.2	0.86	(0.56, 1.34)	0.96	(0.60, 1.54)	0.70	(0.41, 1.20)
Ex2+159C>T	CT	188	10.8	1.02	(0.70, 1.47)	1.06	(0.71, 1.57)	0.87	(0.56, 1.35)
	TT	6	0.4	0.76	(0.14, 4.17)	0.82	(0.13, 5.09)	0.46	(0.08, 2.76)
-3714G>T	GT	194	11.1	1.01	(0.70, 1.45)	1.17	(0.80, 1.73)	0.80	(0.51, 1.24)
	TT	8	0.5	0.74	(0.15, 3.56)	0.92	(0.18, 4.78)	0.48	(0.08, 2.87)

**Haplotype**†									

A-C-G-*		2153	50.9	1.00	(reference)	1.00	(reference)	1.00	(reference)
A-C-G-9		596	14.1	0.92	(0.72, 1.18)	**0.74**	**(0.56, 0.98)**	**0.70**	**(0.52, 0.94)**
A-T-T-*		202	4.8	1.15	(0.76, 1.73)	1.23	(0.79, 1.93)	0.94	(0.57, 1.53)
G-C-G-*		592	14	1.05	(0.82, 1.36)	1.12	(0.85, 1.47)	0.91	(0.68, 1.23)
G-C-G-9		456	10.8	1.07	(0.81, 1.43)	1.12	(0.82, 1.53)	0.96	(0.69, 1.34)

* = an allele other than the SLC6A3*9 allele. These are largely (98.1%) the SLC6A3*10 VNTR allele.

† Haplotype analyses were conducted among non-Hispanic Caucasians

‡ OR estimated using conditional logistic regression, conditioning on age, sex, current smoking status, number of cigarettes smoked

We found no evidence of interaction between polymorphisms at *SLC6A3 *and *DRD2/ANKK1*. Among individuals with the *DRD2/ANKK1 *haplotype (T-C-T-A) previously associated with obesity [25], no marked increase in effect of the *SLC6A3 *VNTR on obesity was observed (** = reference; OR_*9 _= 0.89, 95% CI 0.54–1.46; OR_99 _= 0.54, 95% CI 0.23–1.33, p-value for interaction = 0.62).

Analyses restricted to non-Hispanic Caucasians were consistent with the findings above (data not shown).

We did not observe any significant associations of the *SLC6A3 *polymorphisms with any of the smoking outcomes (number cigarettes per day, smoking duration, age at initiation, pack years) or alcohol consumption.

## Discussion

We report a protective effect of the 9 allele of the *SLC6A3 *VNTR on overweight and obesity and a novel association between the presence of the G allele of *SLC6A3 *Ex9-55A>G polymorphism and being underweight. These results support the role of genetic variation at the dopamine transporter locus as modifiers of body weight and extend our earlier findings that polymorphisms in the D2 dopamine receptor are associated with both cigarette smoking and obesity [25,26], suggesting that multiple genes in the dopaminergic system influence these behaviors.

Dopaminergic function may modulate reward from both food and drugs of abuse. This reward deficiency hypothesis (constitutional anhedonia predisposing individuals to reliance on extrinsic stimuli such as food, nicotine or alcohol) provides the dominant framework in molecular genetic studies of dopaminergic function and its effects on drug addiction (e.g., [30,31]). This model has also motivated, in part, interpretation of neuroimaging based studies, which have provided molecular and functional neuroanatomical evidence on the relationship between dopaminergic function and drug addiction or obesity [32].

The dopamine transporter (DAT1, *SLC6A3*) is responsible for the reuptake of dopamine into the presynaptic dopaminergic cell from the dopamine synapse. The *SLC6A3 *VNTR polymorphism has been considered to be functional based on a relation of the repeats to regulation of promoter activity. *in vitro *and *ex vivo *studies involving human tissues have shown that the SLC6A3*10 allele has been associated with increased concentrations of DAT protein, when compared with the SLC6A3*9 allele [33-36], although neuroimaging approaches involving indirect estimation of striatal DAT levels by antagonist displacement and single photon emission computed tomography (SPECT) have not revealed consistent differences in DAT levels between 9 and 10 repeats [37-40]. Mechanistically, increasing the number of variable repeats appears to result in higher concentrations of the DAT protein. This, in turn, may lead to higher dopamine transporter binding and more efficient dopamine clearance, resulting in lower postsynaptic concentrations of dopamine. Presence of "faster" dopamine reuptake, as seen with the 10 repeat allele, may affect the reinforcing value of food.

This present study supports the association between the presence of the 9 allele of *SLC6A3 *VNTR and decreased risk of obesity. Our findings are supported by earlier work of Epstein *et al*, who first reported the association of the 10 allele and obesity in African American smokers [24]. A recent study evaluating polymorphisms in genes involved in dopamine availability in a UK cohort of Caucasian females did not find this association; however, the study was not sufficiently large enough to detect an effect of the size that we detected at the *SLC6A3 *VNTR [41]. Another recent study did not report an association with the *SLC6A3 *VNTR and BMI, but this study was based on an adolescent cohort, which had large demographic differences from our study [42]. Also, although recent genome wide association studies have identified common genetic variation in the melanocortin 4 receptor (*MC4R*) and *FTO *(the fat mass and obesity associated) genes, an association with the dopamine transporter was not reported [7-9,11,12]. However, it is important to stress that genome-wide association studies do not capture all genetic variation in the human genome (for example, variants with MAF < 5% are often completely absent); therefore, candidate gene studies will continue to be important to verify and provide more detailed analyses of biologically plausible candidates [43].

This present study also supports an association between presence of the G allele of *SLC6A3 *Ex9-55A>G polymorphism and being underweight. Although this relationship is statistically striking, further study is required to validate this finding. However, dopamine dysregulation is present in anorexia nervosa. For example, increased central dopaminergic activity is observed in weight-recovered anorectics compared to controls [44], and dopamine D2 and D4 receptor polymorphisms have been associated with anorexia [45,46]. At present, it is unknown if the *SLC6A3 *Ex9-55A>G polymorphism represents a functional variant. It is possible that this variant influences transcription, transcript stability or translation [47] or that it is in linkage disequilibrium with a functional polymorphism; exon 9 and the VNTR polymorphism have been reported to be in linkage disequilibrium [48].

Smoking and being overweight are related [49] and both increase the risk of death from the major causes of mortality including heart disease, diabetes and cancer [50-53]. As expected, we saw a significant difference in smoking status by BMI, with former smokers more likely to be obese than their smoking counterparts. This association has been previously described, as well as weight gain after smoking cessation [54,55]. Previous studies have shown that this association may be due to a relationship between food reinforcement and dopamine genotypes in smokers, as smoking cessation decreases the activation of the reward pathway, resulting in the substitution of food for cigarettes [56]. Earlier work suggested an interaction of the dopamine receptor and transporter on both smoking and cessation outcome [57,58]. As smoking and being overweight are related [49,54,55] and the same genetic polymorphisms involved in the dopaminergic reward pathway that regulates food reward may also influence reward from nicotine, it is reasonable to postulate that *DRD2 *and the dopamine transporter interact to increase their effects on BMI. Although we failed to observe formal statistical interaction (supraadditive or multiplicative effect) of the two genes, the additive effects observed are consistent with effects of both genes on obesity.

It is important to note some limitations with this study. We relied on self-report data for height and weight to calculate BMI. This type of self-report data has an inherent degree of misclassification, which can bias results towards the null. In order to minimize this misclassification error, we categorized BMI by established, broad categories. Use of this classification is widely accepted in the literature but does slightly reduce power; the original continuous BMI variable would have provided a slightly more powerful test for association with the genetic polymorphisms. Also, although our study size was substantial for a study of this type, reliable detection of interaction or subgroup effects generally requires much larger study samples or consortia.

## Conclusion

This study has identified an association of a functional polymorphism at a key dopaminergic locus, the dopamine transporter (*SLC6A3*), and BMI. The direction of this allelic effect is consistent with prior functional studies, suggesting that reduced reuptake of dopamine is protective against overweight and obesity, as well as increases in BMI. Further study of the relationships between the dopaminergic system and BMI is warranted, including taking into account sensitivity to reward as a factor influencing both diet and exercise [59].

## Competing interests

The authors declare that they have no competing interests.

## Authors' contributions

EMA was involved in conception and design of the study, acquisition of data, analysis and interpretation of data, and drafting of the manuscript. LMM and AWB were involved in conception and design of the study, acquisition of data, statistical analysis, analysis and interpretation of data, supervision of the study, and critical review of the manuscript. SSW was involved in conception and design of the study, obtaining funding, acquisition of data, statistical analysis, administrative, technical or material support, and supervision of the study. NC was involved in statistical analysis, analysis and interpretation of data, and critical revision of the manuscript for important intellectual content. PK was involved in analysis and interpretation of data, administrative, technical or material support, and critical revision of the manuscript for important intellectual content. MY and RBH provided administrative, technical or material support and manuscript review. SJC was involved in analysis and interpretation of data and provided administrative, technical or material support. NEC was involved in conception and design of the study, obtaining funding, acquisition of data, analysis and interpretation of data, supervision of the study, administrative, technical or material support, drafting the manuscript and critical revision of the manuscript for important intellectual content. All authors read and approved the final manuscript.

## Pre-publication history

The pre-publication history for this paper can be accessed here:



## Supplementary Material

Additional file 1 **Supplemental tables.** This file includes Supplemental tables referenced in the main manuscript, including Supplemental table 1: Selected polymorphisms at SLC6A3, DRD2 and ANKK1, Supplemental table 2: SLC6A3 polymorphisms and haplotypes by the slope of weight change. Click here for file
