# Supplementary material for: SLC6A3 and body mass index in the Prostate, Lung, Colorectal and Ovarian Cancer Screening Trial
Source: BMC Med Genet. 2009 Jan 30;10:9. doi: 10.1186/1471-2350-10-9 (PMC2640369; doi:10.1186/1471-2350-10-9)
Supplement: Additional file 1 — Supplemental tables. This file includes Supplemental tables referenced in the main manuscript, including Supplemental table 1: Selected polymorphisms at SLC6A3, DRD2 and ANKK1, Supplemental table 2: SLC6A3 polymorphisms and haplotypes by the slope of weight change. [file 1471-2350-10-9-S1.doc]

Supplemental table 1. Selected polymorphisms at *SLC6A3*, *DRD2* and *ANKK1*

| **Gene** | **Name** | **Chromosomal**  **Location** | **SNP rs#** | **Polymorphism** | **Amino Acid Change** | **Minor Allele Frequency** |
| --- | --- | --- | --- | --- | --- | --- |
| *SLC6A3* | Dopamine transporter | 5p15.3 | rs6350 | Ex2+159C>T | N38N | 0.276 |
|  |  |  | rs6413429 | -3714G>T |  | 0.052 |
|  |  |  | rs6347 | Ex9-55A>G | S405S | 0.060 |
|  |  |  |  | 3’UTR 40-bp variable number of tandem repeats |  | 0.280* |
| *ANKK1* | Ankyrin repeat and kinase domain containing 1 | 11q23 | rs1800497 | Ex8-313G>A | E713K | 0.250 |
| *DRD2* | Dopamine receptor D2 | 11q23 | rs6277 | Ex7+147C>T | P319P | 0.268 |
|  |  |  | rs1076560 | IVS6-83G>T |  | 0.195 |
|  |  |  | rs1799978 | -50977A>G |  | 0.103 |

*Frequency of the 9 allele

Supplemental table 2. SLC6A3 polymorphisms and haplotypes by the slope of weight change

|  |  |  |  | **Weight change slope (kg/m^2^ per 10 years)^§^** | | | | | |
| --- | --- | --- | --- | --- | --- | --- | --- | --- | --- |
|  |  |  |  | **0.31-0.70 kg/m^2^ (N=1170) vs. ≤ 0.30 kg/m^2^ (N=1038)** | | **0.71-1.20 kg/m^2^ (N=1040) vs. ≤ 0.30 kg/m^2^ (N=1038)** | | **> 1.20 kg/m^2^ (N=966) vs. ≤ 0.30 kg/m^2^ (N=1038)** | |
| **Polymorphism** |  | **N** | **G Freq (%)** | **OR**‡ | **(95% CI)** | **OR**‡ | **(95% CI)** | **OR**‡ | **(95% CI)** |
| SLC6A3 VNTR | *9 | 724 | 39.9 | 0.97 | (0.76, 1.23) | 0.88 | (0.68, 1.13) | 0.88 | (0.67, 1.14) |
|  | 99 | 119 | 6.5 | 1.07 | (0.68, 1.68) | 0.76 | (0.46, 1.26) | 0.58 | (0.33, 1.01) |
| Ex9-55A>G | AG | 711 | 39.0 | 1.22 | (0.96, 1.56) | **1.32** | **(1.02, 1.70)** | 1.04 | (0.80, 1.36) |
|  | GG | 138 | 7.2 | 0.92 | (0.58, 1.44) | 1.07 | (0.68, 1.69) | 0.66 | (0.39, 1.12) |
| Ex2+159C>T | CT | 188 | 10.8 | 1.03 | (0.71, 1.48) | 0.90 | (0.62, 1.32) | 0.86 | (0.57, 1.31) |
|  | TT | 6 | 0.4 | 0.91 | (0.17, 4.87) | 0.66 | (0.11, 4.09) | 0.48 | (0.08, 3.00) |
| -3714G>T | GT | 194 | 11.1 | 1.02 | (0.71, 1.46) | 0.94 | (0.65, 1.37) | 0.82 | (0.54, 1.25) |
|  | TT | 8 | 0.5 | 0.94 | (0.20, 4.51) | 0.77 | (0.15, 3.94) | 0.53 | (0.09, 3.36) |
| **Haplotype**† |  |  |  |  |  |  |  |  |  |
| A-C-G-* |  | 2153 | 50.9 | 1.00 | (reference) | 1.00 | (reference) | 1.00 | (reference) |
| A-C-G-9 |  | 596 | 14.1 | 0.92 | (0.72, 1.18) | **0.69** | **(0.52, 0.90)** | **0.70** | **(0.53, 0.93)** |
| A-T-T-* |  | 202 | 4.8 | 1.18 | (0.79, 1.77) | 0.87 | (0.56, 1.34) | 0.93 | (0.58, 1.48) |
| G-C-G-* |  | 592 | 14 | 1.04 | (0.80, 1.35) | 1.10 | (0.85, 1.43) | 0.85 | (0.64, 1.14) |
| G-C-G-9 |  | 456 | 10.8 | 1.19 | (0.89, 1.58) | 1.10 | (0.82, 1.49) | 0.84 | (0.61, 1.17) |

*****= an allele other than the SLC6A3*9 allele. These are largely (98.1%) the SLC6A3*10 VNTR allele.

† Haplotype analyses were conducted among non-Hispanic Caucasians

‡ OR estimated using conditional logistic regression, conditioning on age, sex, current smoking status, number of cigarettes smoked.

**^§^** Change in BMI slope corresponds to change in weight slope, as height was only reported once at the time of baseline questionnaire
